# Real World Outcomes and Hepatotoxicity of Infliximab in the Treatment of Steroid-Refractory Immune-Related Adverse Events

**DOI:** 10.3390/curroncol28030201

**Published:** 2021-06-11

**Authors:** Daniel V. Araujo, Thiago Pimentel Muniz, Anjie Yang, Sareh Keshavarzi, Hadas Sorotsky, Marcus O. Butler, Samuel Saibil, Anna Spreafico, David Hogg

**Affiliations:** 1 Department of Medical Oncology and Hematology, Princess Margaret Cancer Center, Toronto, ON M5G 1X6, Canada; thiago.muniz@uhn.ca (T.P.M.); hsorotsky@gmail.com (H.S.); Marcus.Butler@uhn.ca (M.O.B.); sam.saibil@uhn.ca (S.S.); anna.spreafico@uhn.ca (A.S.); david.hogg@uhn.ca (D.H.); 2 Department of Medical Oncology, Hospital de Base, Sao Jose do Rio Preto 15090 000, Brazil; 3 Department of Pharmacy, University Health Network, Toronto, ON M5G 2M9, Canada; anjie.yang@uhn.ca; 4 Department of Biostatistics, Princess Margaret Cancer Center, Toronto, ON M5G 2M9, Canada; Sareh.Keshavarzi@uhnresearch.ca; 5 Biostatistics Division, Dalla Lana School of Public Health, University of Toronto, Toronto, ON M5T 3M7, Canada; 6 Cancer Center, Chaim Sheba Medical Center at Tel HaShomer, Ramat Gan 52621, Israel

**Keywords:** infliximab, immunotherapy, toxicity, hepatotoxicity, immune-checkpoint inhibitors

## Abstract

Background and aims: Current guidelines state that infliximab is contraindicated for the treatment of immune checkpoint inhibitor-related hepatitis (ir-hepatitis) due to the risk of inducing further liver damage. As this recommendation is largely based on the use of infliximab for rheumatologic diseases, we evaluated the efficacy and hepatotoxicity of infliximab in patients with steroid-refractory immune-related adverse events (irAEs). Methods: We retrospectively reviewed consecutive patients treated with infliximab for irAEs at Princess Margaret Cancer Centre. To assess hepatotoxicity, we compared the mean value of ALT, AST, and total bilirubin (BT) before and after infliximab treatment. We used logistic regression to assess factors associated with infliximab efficacy. Results: Between January 2010 and February 2019, 56 patients were identified. The median age of the patients was 63 (27–84) years. Colitis was the most frequent toxicity (66%), followed by pneumonitis (11%). Infliximab was used to treat ir-hepatitis in one patient. The median number of infliximab doses was 1 (1–3) and led to toxicity resolution in 43 (76%) patients. The mean ALT, AST, and BT levels before and after infliximab treatment were not statistically different. The patient treated for ir-hepatitis had a complete recovery, with no incremental liver toxicity. Conclusions: In this dose-limited setting, infliximab was effective in resolving irAEs and did not induce hepatotoxicity.

## 1. Introduction

Immune checkpoint inhibitors (ICI), such as anti-programmed cell death 1 (anti-PD1), anti-programmed cell death-ligand 1 (anti-PD-L1), and anti-cytotoxic T-Lymphocyte associated protein 4 (anti-CTLA-4) agents, act by disrupting inhibitory mechanisms of T-cell activation facilitating T-cell mediated cytotoxicity to cancer cells [1]. ICIs are administered either as monotherapy or a combination therapy across a variety of cancer types. Despite being safer than cytotoxic chemotherapy overall, ICIs frequently induce immune-related adverse events (irAEs) of varying severity. While most patients with severe irAEs achieve the complete resolution of their toxicities with the use of steroids, a smaller proportion are steroid-refractory and require additional immunosuppressive agents, including the anti-tumor necrosis factor alfa agent infliximab [2,3]. Infliximab is currently approved for the treatment of various non-cancerous autoimmune and inflammatory conditions such as inflammatory bowel diseases (IBD) [4] and psoriasis [5]. Although infliximab is recommended for most steroid-refractory irAEs, from relatively common conditions such as ir-colitis to other rarer irAEs [6], current guidelines recommend against its use for immune-related hepatitis (ir-hepatitis) [2,3,7]. This tenet originates from reports of infliximab-induced hepatotoxicity in patients receiving infliximab as a treatment for rheumatologic disorders or IBD [8,9]. In those reports, most cases of hepatitis occurred following ongoing exposure to infliximab, in contrast to a single dose of infliximab that is commonly used in managing irAEs.

In this work, we examined the efficacy and hepatotoxicity of infliximab in a single institution series of cancer patients with steroid-refractory irAEs, with the aim to evaluate a possible revision of recommendations regarding the use of infliximab as a treatment for ir-hepatitis.

## 2. Materials and Methods

Following Research Ethics Board approval, we retrospectively identified consecutive patients treated with infliximab for irAEs deemed to be steroid refractory by the attending physician. Patients received infliximab at Princess Margaret Cancer Centre between January 2010 and February 2019. Data regarding tumor type, toxicity, ICI regimen at irAE onset (monotherapy vs. combination of 2 ICIs), toxicity resolution, tumor response to ICI, and survival were collected. Demographic characteristics were summarized as means, medians, and proportions. We assessed hepatotoxicity by comparing mean values of ALT, AST, and total bilirubin (BT) between 0–4 weeks before and after infliximab therapy. When values were not available within 4 weeks, the nearest report was utilized. The Wilcoxon signed rank test was used to compare mean values. We used a logistic regression to investigate characteristics associated with irAE resolution. We performed multivariable analysis to adjust for potential confounders. Treatment efficacy assessed at the time of ICI toxicity onset was recorded as response rate (RR) per attending physician’s assessment and categorized as complete response (CR), partial response (PR), stable disease (SD), or progressive disease (PD). Overall survival (OS) was calculated from the date of the first infliximab dose to date of death or last follow-up. All of the statistical analyses were performed in R (version 3.6.3, R Foundation for Statistical Computing, https://www.R-project.org/ accessed on 8 March 2021), and a two-sided alpha level of 0.05 was used for determining statistical significance.

## 3. Results

We identified 56 patients who were included in this analysis. Table 1 summarizes patients’ demographic characteristics. The median age was 63 years (27–84) and 38 (68%) patients were male. The cancer types included melanoma in 35 (62%), renal cell carcinoma (RCC) in 5 (9%), and non-small cell lung cancer (NSCLC) in 4 (7%) patients. At the time of the irAE onset, 25 (45%) patients were receiving combination immunotherapy, of whom 19 (76%) were an anti-PD-1 with an anti-CTLA-4 agent. Twenty-seven (48%) patients were receiving monotherapy, of whom 13 (48%) were on anti-PD1 and 11 (40%) were being treated with ipilimumab (anti-CTLA-4). Another 4 (7%) patients were being treated within blinded randomized clinical trials and had not been unblinded by a data cutoff. The most frequent toxicities treated with infliximab were colitis (37 cases; 66.1%) and pneumonitis (6 cases; 10.7%). One patient (1.7%) with ir-hepatitis received infliximab.

The median number of infliximab treatments was one (1–3). Infliximab treatment led to the resolution of irAEs in 43 (76%) patients. Fourteen patients (25%) required more than one dose of infliximab, which were separated by a median of 40.5 (12–867) days. After multivariable adjustment, colitis was more likely to respond to infliximab compared to all of the other irAEs combined (OR = 6.73, 95% CI 1.56–29, *p* = 0.011). The tumor type (melanoma vs other cancers; OR = 0.93, 95% CI 0.21–4.02, *p* = 0.92) and combination vs monotherapy ICI treatment (OR = 1.97, 95% CI 0.48–8.11, *p* = 0.64) did not affect the likelihood of an infliximab response (Table 2).

**Figure 1:**
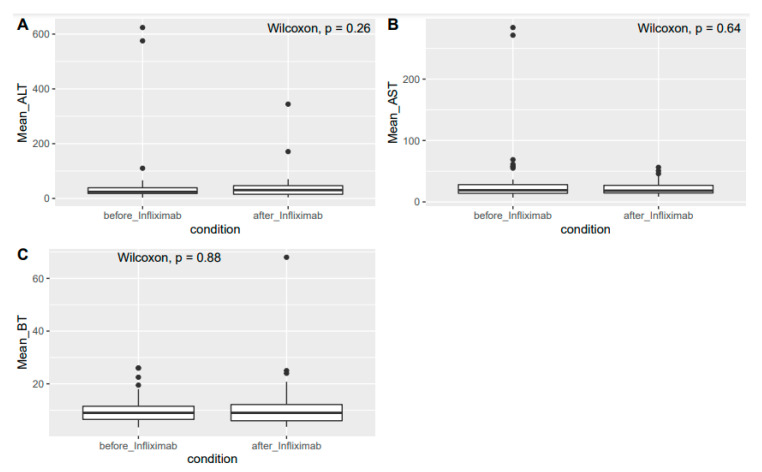
(**A**) Mean values of AST before and after infliximab. (**B**) Mean values of ALT before and after infliximab. (**C**) Mean values of TBILI before and after infliximab.

There was no statistical difference between the mean values of AST, ALT, and BT before and after infliximab treatment (AST: 31.7 vs. 21.2 U/L, *p* = 0.5; ALT: 49 vs. 39.1 U/L, *p* = 0.2; and BT: 9.9 vs. 10.9 μmol/L, *p* = 0.8, respectively, Figure 1). A 27-year-old man with metastatic melanoma treated with the combination of an anti-CTLA-4 and an anti-PD1 agent developed steroid-refractory ir-hepatitis and was treated with one cycle of infliximab at the standard dose of 5 mg/kg, achieving the normalization of transaminases. The patient did not experience recrudescence of hepatitis, further liver toxicity, or the occurrence of additional irAEs after infliximab treatment. Other than steroids, the patient did not receive any other immunosuppressive therapy before infliximab.

In our cohort, the recurrence of an irAE (either the same or another irAE) occurred in 17 (31%) patients, and 46 (82%) patients did not resume ICI treatment post-infliximab treatment (Table 1). In regard to the efficacy of the ICI treatment being used at the time of toxicity, 50 patients were available for assessment. The best response was CR in 4 (7%), PR in 12 (22%), SD in 13 (24%), and PD in 22 (38%) patients. The median OS from the first infliximab dose was 13 months (95% CI 7.3–19.3).

## 4. Discussion

In our study, infliximab was associated with the resolution of 76% of steroid-refractory irAEs in a real-world setting at an academic institution. The median number of infliximab infusions was one, which is in keeping with clinical experience [10]. Within this dose-limited scenario (median number of doses was one), we did not observe significant changes between the mean AST, ALT, and BT values before and after infliximab treatment. The patient with steroid-refractory ir-hepatitis that was treated with infliximab responded well to treatment and his hepatitis resolved.

To the best of our knowledge, this is the first study that systematically examined the hepatotoxicity of infliximab in the treatment of steroid-refractory irAEs. Infliximab-induced hepatotoxicity has been described in patients with primary autoimmune conditions or IBD [8,9]. However, those patients were either treated on an ongoing basis and received multiple infliximab treatments over time (the median number of infliximab doses before hepatitis development was four) or were treated at a higher dose than the standard 5 mg/kg typically used in clinical practice for steroid-refractory irAEs [8,11,12]. Moreover, the usual treatment of infliximab-induced-hepatoxicity includes high-dose steroids, and most patients with ICI-induced irAEs are already receiving these drugs [12,13].

Infliximab has been previously used to treat autoimmune hepatitis refractory to azathioprine in a cohort of 11 patients leading to a decrease in transaminases, without inducing any additional liver toxicity [14]. In addition, a recent case report by Corrigan et al. described the use of infliximab to treat a patient with metastatic melanoma who developed ir-hepatitis refractory to both steroids and treatment with mycophenolate mofetil (MMF) [15]. After infliximab treatment, hepatitis was resolved, and no liver-toxicity was induced.

There is no published evidence that infliximab induces hepatotoxicity in a dose-limited setting in oncology patients, or that infliximab may aggravate steroid-refractory ir-hepatitis. On the contrary, the recommendation to avoid infliximab in patients with ir-hepatitis may compromise the optimal treatment of this irAE. The current immunosuppressants of choice for ir-hepatitis—MMF and azathioprine—require tapering, which may delay the re-initiation of ICI treatment for metastatic disease, thus potentially compromising the treatment’s effectiveness. In contrast, infliximab is associated with a rapid kinetics of response and may allow for the earlier re-initiation of ICI treatment [10]. Nevertheless, re-initiation of ICI after an irAE carries a 28.8% chance of recurrence of the same irAE or may trigger the occurrence of a different one in 4.4% of cases [16].

In terms of ICI effectiveness, our data is consistent with a report by Burdett et al. in which a cohort of 19 patients required additional immunosuppression (refractory to steroids) for irAEs and an overall response rate to ICI of 35% and a median OS of 9.4 months was found [17]. Overall, these results are inferior to those reported in the pivotal trials of ICI for melanoma [18], NSCLC [19,20], and RCC [21,22]. The heterogeneity of our cohort, both in respect to different tumor types and different stages, as well as the small number of patients included, may explain some of the observed discrepancies. While the occurrence of irAEs in general has been found to correlate with increased survival, most patients included in these analyses developed mild toxicities [23]. The potential detrimental effects of long-term/stronger immunosuppression, including infliximab, in patients who developed ICI-related toxicities are yet to be determined and should be evaluated in larger datasets [24].

The limitations of this work include its retrospective nature, the absence of steroid-refractory disease confirmation in all patients (e.g., colonoscopy and biopsy in colitis, or bronchoscopy and BAL in pneumonitis), and the low rate of ir-hepatitis treated with infliximab (one patient). Nonetheless, to date, this is the first study systematically assessing the hepatoxicity of infliximab in the treatment of irAEs and the largest cohort assessing outcomes of patients treated with infliximab in the setting of ICI-induced steroid-refractory irAEs.

## 5. Conclusions

In our cohort, infliximab was associated with a high rate of resolution of irAEs and was not associated with hepatoxicity development. A patient with ir-hepatitis that was treated with infliximab had his toxicity resolved with no recurrence. Infliximab may be an option for the treatment of ir-hepatitis and should be tested in a randomized trial to determine its efficacy and safety in the dose-limited context of ICI-related toxicity.

## Figures and Tables

**Table 1:** Characteristics of patients treated with infliximab. * Other cancer types are HNSCC, Uveal melanoma, esophageal-gastric adenocarcinoma, urothelial carcinoma, pancreas, breast. ** Participating in clinical trials randomizing to receive combination versus ICI monotherapy. *** Participating in clinical trials randomizing to receive either anti-CTLA-4 or anti-PD1. **** Other toxicities are erythema multiform dermatitis, arthritis, type 1 diabetes, Stevens–Johnson syndrome, rash, bullous pemphigoid, enteritis, and cytokine release syndrome. NSCLC = non-small cell lung cancer; RCC = renal cell carcinoma.

N	56
Age—mean (SD)	62.9 (SD 12)
Gender—*n* (%)	
Female	18 (32%)
Male	38 (68%)
Type of Cancer—*n* (%)	
Melanoma	35 (62%)
NSCLC	4 (7%)
RCC	5 (9%)
Others *	12 (22%)
Combination therapy at time of toxicity—*n* (%)	
No	27 (48%)
Yes	25 (45%)
Unknown **	4 (7%)
ICI monotherapy—*n* (%)	
Anti-CTLA4	11 (40.8%)
Anti-PD1	13 (48.1%)
Off ICI	1 (3.7%)
Unknown ***	2 (7.4%)
ICI Combination—*n* (%)	
Anti-CTLA4 + Anti-PD1	19 (76%)
Other	6 (24%)
Type of Toxicity—*n* (%)	
Colitis	37 (66.1%)
Hepatitis	1 (1.8%)
Myocarditis	2 (3.6%)
Pneumonitis	6 (10.7%)
Others ****	10 (17.8%)
Multiple toxicities at Onset—*n* (%)	
No	45 (80%)
Yes	11 (20%)
Line of ICI treatment—*n* (%)	
Adjuvant	5 (9%)
1st	29 (52%)
2nd	13 (23%)
3rd	6 (11%)
≥4th	3 (5%)
Best response to ICI that induced toxicity—*n* (%)	
CR	4 (7%)
PR	12 (21%)
SD	13 (23%)
PD	22 (39%)
N/A	5 (9%)
Outcome of toxicity—*n* (%)	
Not resolved	13 (24%)
Resolved	42 (76%)
Missing	1
Resumed ICI—*n* (%)	
No	46 (82%)
Yes	10 (18%)
Recrudescence of Toxicity—*n* (%)	
No	38 (69%)
Yes	17 (31%)

**Table 2:** Characteristics associated with the resolution of an irAE post-infliximab.

Variable	Resolved	Not Resolved	Univariable			Multivariable		
			OR	95% CI	*p*	OR	95% CI	*p*
Type of toxicity								
Colitis	32	5	5.12	1.36–19.24	0.016	6.73	1.56–29.04	0.011
Others	10	8	Reference			Reference		
Type of cancer								
Melanoma	7	28	1.71	0.48–6.07	0.4	0.93	0.21–4.02	0.92
Others	6	14	Reference			Reference		
Combo ICI at time of toxicity					0.73			0.64
Yes	5	20	1.68	0.47–6.07		1.97	0.48–8.11	
No	8	19	Reference	N/A		Reference		

## Data Availability

The datasets used and/or analysed during the current study are available from the corresponding author on reasonable request.
